# Adaptation of the Kirkstall QV600 LLI Microfluidics System for the Study of Gastrointestinal Absorption by Mass Spectrometry Imaging and LC-MS/MS

**DOI:** 10.3390/pharmaceutics14020364

**Published:** 2022-02-05

**Authors:** Chloe E. Spencer, Stephen Rumbelow, Steve Mellor, Catherine J. Duckett, Malcolm R. Clench

**Affiliations:** 1 Centre for Mass Spectrometry Imaging, Biomolecular Sciences Research Centre, Sheffield Hallam University, Howard Street, Sheffield S1 1WB, UK; chloe.e.spencer@student.shu.ac.uk (C.E.S.); c.duckett@shu.ac.uk (C.J.D.); 2 CRODA Inc. (B88), New Castle, DE 19720, USA; stephen.rumbelow@croda.com; 3 CRODA Europe Ltd., Cowick DN14 9AA, UK; steve.mellor@croda.com

**Keywords:** mass spectrometry imaging, microfluidics, Atorvastatin, polysorbate 80

## Abstract

Absorption studies on oral drugs can be difficult due to the challenge of replicating the complex structure and environment of the GI tract. Drug absorption studies can be conducted using in vivo and ex vivo animal tissue or animal-free techniques. These studies typically involve the use of Caco-2 cells. However, Caco-2 cells do not incorporate all the cell types found in intestinal tissue and lack P450 metabolizing enzymes. The QV600 LLI system is a microfluidics system designed for use with cell culture. Here, it has been adapted to house appropriate sections of ex vivo porcine tissue to act as a system that models the duodenum section of the small intestine. A pH regulated solution of Atorvastatin was flowed over the apical layer of the GI tissue and a nutrient solution flowed over the basal layer of the tissue to maintain tissue viability. The tissue samples were snap-frozen, cryosectioned, and imaged using MALDI Mass Spectrometry Imaging (MSI). A proof-of-concept study on the effect of excipients on absorption was conducted. Different concentrations of the solubilizing agent were added to the donor circuit of the QV600 LLI. The amount of Atorvastatin in the acceptor circuit was determined to study the effect of the excipient on the amount of drug that had permeated through the tissue. Using these data, Papp, pig values were calculated and compared with the literature.

## 1. Introduction

Within the pharmaceutical industry, a variety of dosage forms have been developed for the purpose of administering medication. Amongst those, oral administration remains the most popular route with patients [1,2]. The ease of delivery and non-invasive nature of oral drug delivery contribute to the high level of patient compliance experienced with this route. However, the study of drug absorption within the gastrointestinal (GI) tract is highly complex.

The GI tract is an enclosed structure within the body that demonstrates fluctuating conditions as you move along its length. Gastric acid secreted within the stomach has an extremely low pH which creates a highly acidic environment typically ranging between 1–3 [3]. In the movement from the stomach to the duodenum of the small intestine, the pH rapidly increases to pH 6 [4]. During the passage to the large intestine, a neutral pH is reached. With varying acidic environments, differing structures are needed to withstand such extreme environments. Specialized cells such as foveolar cells are found exclusively in the stomach [3]. Foveolar cells are essential to withstanding the extreme acidity within the stomach; they are responsible for the production of mucus which lines the gastric mucosa and provides protection from the gastric acid [3]. In addition, every organ in the GI tract has a different function which is facilitated by its structure and unique features. Both the stomach and the small intestine share the same function which is to break down food. However, both organs have a different tissue structure. The stomach has a unique muscle layer known as the oblique layer that breaks food down by grinding with digestive juices [3]. The small intestine on the other hand, lacks this specialized muscle layer but instead utilizes bile and enzymes to break food down for absorption. Within this single organ, three different sections exist: the duodenum, jejunum, and ileum. Each section has slight variations in structure which are tailored to suit its function. The duodenum is the only section in the small intestine which has Brunner’s glands within its submucosa layer; the purpose of these is the secretion of an alkaline fluid which will protect the duodenum from the highly acidic contents that enter from the stomach while they’re being neutralized [5]. The jejunum and ileum both contain structures, known as plicae circulares, which are muscular flaps that increase surface area and improve the rate of nutrient absorption [6]. These structures are not found in the first section of the small intestine. Thus, the varying structures and extreme conditions experienced within the several organs that form the GI tract are difficult to accurately replicate, thereby hindering the development of alternatives to animal models for the study of drug absorption and excipient performance [7].

The need for realistic organ and tissue models is becoming more apparent as animal testing for research purposes is losing societal acceptance [8]. Under the Cosmetic Directive 76/768/EEC, the EU prohibited testing cosmetic products and cosmetic ingredients on animals in 2004 and 2009, respectively. In 2013, a complete EU ban on animal testing for cosmetics came into full force which prohibits the marketing of any cosmetics in the EU that have been tested on animals. This movement has undoubtedly sparked concern within the pharmaceutical research industry with a significant rise in the amount of research studies that use animal testing alternatives [8]. Well-established alternatives include the use of cultured cells and ex vivo tissue housed in microfluidic systems [9].

For oral drug absorption and permeation research, a common cell line used are colorectal adenocarcinoma (Caco-2) cells. These cells are typically cultured within microfluidic devices or systems in order to study drug permeation and absorption. Giusti et al. has successfully reported the use of Caco-2 cells in a membrane bioreactor that was influenced by the QV600 LLI produced by Kirkstall Ltd. (York, UK) [10]. The feasibility of the membrane bioreactor as a dynamic intestinal barrier model when combined with Caco-2 cells was successfully demonstrated [10]. Although this cell line is considered to be a gold standard, it lacks drug metabolizing P450 enzymes which are essential to accurately represent the absorption of drug classes such as statins [1,11]. Whilst P450 enzyme activity largely occurs in the liver, cytochrome P450 can be found in the epithelial cells of the GI tract [9]. Thus, monocultured cells are not the most suitable system for the study of every drug class.

The major benefit of using ex vivo intestinal tissue is that it maintains important morphological and physiological features of the intestine which aid drug absorption; circular folds in the lumen of the small intestine known as plicae circulares increase the surface area in the intestine and thus enhance drug absorption. A popular microfluidic system used to house ex vivo intestinal tissue is the Ussing chamber. Arnold et al. conducted a study focused on combining porcine intestinal tissue with the Ussing chamber to evaluate drug transport and absorption [12]. The study was successful and demonstrated that ex vivo porcine tissue was compatible with the Ussing chamber system. A limitation of this system is that the removal of the muscle-serosal layer (required for its usage) is complex and labor intensive [13,14]. Additionally, the classic Ussing chamber model doesn’t allow the simultaneous analysis of several tissue segments unlike more recently developed multichannel Ussing systems (although these are expensive). The viability of ex vivo small intestine tissue is limited up to 3 h which makes this technique ideal for drug absorption and permeation studies as drug absorption in the small intestine is rapid. However, this makes ex vivo models unsuitable for long-term exposure studies, unlike cell culture.

Three-dimensional cell models, such as organoids and organ-on-a-chip, combine some of the advantages of 2D cultured cells and ex vivo tissue while eliminating some associated limitations. Organoids are 3D structures that have been derived from patient stem cells or biopsies that can be grown to mimic a particular organ. Organoids contain significantly more morphological and physiological features of an organ than a 2D cell line, which is the advantage that ex vivo tissue has over 2D cell lines. In addition, organoids, have an exceptional viability window in comparison to ex vivo tissue. Wetering et al., report a healthy tissue-derived organoid to be viable after 6 days of drug incubation [15]. This is significantly longer than the viability of ex vivo tissue. Similarly, to 2D cell lines, the limitation of 3D cell models is the amount of time required to culture the cells until they are fully differentiated when compared to ex vivo studies. The animal tissue used for such studies can be quickly acquired from abattoirs or in-house laboratory animals on the day of study. Whereas, the culture of cells can take up to a number weeks; caco-2 cell culture requires 21 days to demonstrate characteristics of the small intestine, as an example [16]. Organ-on-a-chip technology such as microfluidic intestine chip models, are cells cultured in hollow microchannels within a microfluidic device. Primary human cells are cultured in the device which allows the formation of a structure that closely resembles the 3D architecture of real human organs [17,18]. The miniature design of the device leads to the formation of bubbles which are easily formed but difficult to remove [17]. The presence of bubbles in the device can lead to detachment and damage to the cells [17]. Specialized microengineering skills are needed in addition to equipment such as pumps and cleanrooms, thereby making the model an expensive one [17]. Although relatively more expensive when compared to ex vivo model studies, organ-on-a-chip technology provides the possibility for longer experimentation time and a morphology similar to that of the human organ.

All models discussed play a part in drug absorption studies, each with their own benefits and limitations when compared with another. The choice of model is unique to the individual based upon factors including, but not limited to, funding, available facilities, and the purpose of the experiment.

Regardless of whether in vivo or animal testing is used, the historical gold standard for quantifying oral drugs within tissue are radiolabeling methods such as, quantitative whole-body autoradiography (QWBA) [19,20]. These methods boast a combination of quantitative ability with high resolution imaging capabilities which make it highly suited for the evaluation of drug distribution in tissue. A significant limitation of QWBA is that the experiments are restricted to the imaging and quantitation of a single analyte at a time. In addition to this, the placement of the label can lead to false representation of the drug distribution if incorrectly attached to the metabolite; this would be detrimental to quantitative studies [19,21]. Over relatively recent years, QWBA has been commonly combined with liquid chromatography tandem mass spectrometry (LC-MS/MS) which is a label-free technique that boasts high-resolution quantitative abilities for multiple analytes [22]. The combination of techniques reduces the amount of experiments required and therefore, preserves samples whilst reducing the cost and time spent. Despite the advantages of LC-MS/MS, it cannot be used alone as it lacks imaging capabilities and therefore, provides no spatial information unlike QWBA.

Advancements in mass spectrometry have seen the rise of mass spectrometry imaging (MSI), which is a relatively new label-free method that features the imaging capabilities of QWBA, and the molecular specificity provided by LC-MS/MS. Further development of this method has introduced the combination of MSI with the ionization method, matrix-assisted laser desorption ionization (MALDI) to form MALDI MSI. Benefits of using MALDI include its ability to detect proteins and small drug molecules simultaneously [23]; thus, making the method more than ideal for investigating the spatial distribution of drugs within tissue. Reviews such as those recently published by Spencer et al., highlight the successes of studies that have used 3D tissue models in combination with MALDI MSI for drug-related research [24]. MALDI MSI is becoming an increasingly popular method which addresses the limitations of QWBA while maintaining high-resolution imaging capabilities.

Recent publications have been published that showcase the potential for MALDI MSI to be used for the quantitation of drug distribution within tissue. Russo et al. has successfully demonstrated that quantitative MALDI MSI can be used to quantify and image the spatial distribution of terbinafine hydrochloride in the epidermal region of a full thickness living skin equivalent model [25]. This study used an optimized LC-MS/MS method to corroborate the quantitative abilities of the method which found no statistically significant differences between the data. The successful development and implementation of the quantitative MSI (QMSI) forms a single method that addresses the limitations of both QWBA and LC-MS/MS.

In the study reported here, an existing cell culture system known as the QV600 LLI system (Kirkstall Ltd., York, UK) has been successfully adapted to produce a microfluidics device that is compatible with ex vivo porcine intestinal tissue rather than cells alone. The advantages of adapting the QV600 LLI to house larger samples such as ex vivo tissue is that this makes it a unique, versatile system that can hold ex vivo tissue, human biopsy tissue, and organoids as well as 2D cell lines. This allows contrasting sample types to be directly compared within the same environment and comparative studies to be performed. Another advantage of this system is that many accessories are commercially available to allow for example in situ sampling and closed or open circuit experiments to be performed. In addition to this, multiple chambers can be added to allow for different regions of the intestine to be studied simultaneously. For the purpose of the proof-of-concept study described here, the adaptation of QV600 LLI was developed using ex vivo tissue rather than organoids or any other cell culture as the tissue was readily available, analysis time was significantly shorter and relatively inexpensive which was essential during the development stages. Unlike other commercially available models, the modified system is versatile and can house different sample types; this allows for comparative studies between opposing sample types within the same environment. In addition, its modular nature provides further flexibility in terms of the types of experiments that can be performed

Using the adapted microfluidics device with ex vivo tissue, experiments were performed to study the absorption of statin Atorvastatin through the apical layers of intestinal tissue. MALDI MSI has been applied to show the spatial distribution of Atorvastatin within the tissue. An LC-MS/MS method has been fully optimized to quantify the amount of Atorvastatin in the tissue, basal circuit and other relevant areas to complete a mass balance and thus, aid in the development of the microfluidics device. The LC-MS/MS method was developed in order to act as a validation method that will pave the way for the development of a successful QMSI method in future work.

## 2. Materials and Methods

### 2.1. Materials

Gibco BenchStable DMEM/F12, phosphate-buffered saline (PBS), 2-methylbutane 99+% extra pure, LC-Grade methanol, LC-Grade acetonitrile (ACN), and acetone were purchased from Fisher Scientific Ltd. (Loughborough, UK). Atorvastatin calcium, α-Cyano-4-hydroxycinnamic acid (α-CHCA) and 2,5-Dihydroxybenzoic acid (DHB) were purchased from Sigma Aldrich (Dorset, UK). The deuterated internal standard, Atorvastatin-(anilide ring-d5) calcium salt was purchased from Merck Life Sciences (Dorset, UK). Formic acid 98% was purchased from Scientific Laboratory Supplies (Nottingham, UK). In total, 18.2 MΩ × cm water was collected from an ELGA water purification system (Buckinghamshire, UK). Cryo-M-Bed was purchased from VWR International Ltd. (Lutterworth, UK). Porcine small intestine was provided by R.B Elliott & Son (Chesterfield, UK). Super refined Polysorbate (80) LQ was donated by CRODA (New Castle, DE, USA).

### 2.2. Tissue Collection and Preparation

The duodenum region of the small intestine from an adult pig was collected and prepared within 1 h post-mortem. Using a scalpel, the intestine was cut down its length and opened into a flat sheet. The tissue was then snap-frozen in liquid nitrogen cooled 2-methylbutane and stored at −80 °C until needed. To prepare for drug absorption experiments, the frozen tissue was submerged in PBS until completely thawed. The serosal-muscle layer was carefully removed from the flat sheet of intestinal tissue with a scalpel. A 10 mm diameter tissue punch was then used to remove a disc from the remaining tissue.

### 2.3. Drug Absorption Experiments

The drug absorption experiments were performed in the Quasi Vivo^®^ QV600 LLI system purchased from Kirkstall Ltd. (York, UK). The disc of tissue was adhered to a MilliCell insert with the basal layer facing the mesh. The insert was fitted into the chamber of the QV600 so that the upper circuit flowed above the tissue and the lower circuit flowed below the insert. Three chambers were set up within this system to allow three tissue discs to run simultaneously under the same experimental conditions. The donor circuit was filled with 0.5 mg/mL of Atorvastatin in phosphate-buffered saline and the acceptor circuit was filled with Gibco BenchStable DMEM/F12. All air bubbles were removed while filling. Using a peristaltic pump, the flow rate of the donor and acceptor circuit was set to 0.2 mL/min and 0.1 mL/min, respectively, before the whole system was then transferred to an incubator for 6 h at 37 °C, 5% CO_2_. The circuit fluids were collected, before removing the tissue discs, and stored at 5 °C. The tissue discs were rinsed with PBS before being snap-frozen in liquid nitrogen cooled 2-methylbutane and stored at −80 °C. The whole QV600 system was thoroughly cleaned using 70% methanol in water and then rinsed with PBS. The solutions used to clean the system and the PBS used to rinse the tissue were collected for the mass balance study and stored at 5 °C.

After the initial drug absorption experiment, a series of experiments were performed with varied concentrations of the excipient polysorbate 80 added to the donor circuit alongside 0.5 mg/mL of Atorvastatin. The experiment had otherwise run under the same conditions as described for the initial drug absorption experiment.

### 2.4. Cryosectioning

Tissue discs were transferred to the Leica CM 1950 Cryostat (Leica Microsystems, Milton Keynes, UK). The discs were mounted onto a cork ring using Cryo-M-Bed embedding compound and were allowed to thermally equilibrate for 1 h. The chamber and specimen head temperature were set at −20 °C. Each tissue disc was cryosectioned into 14 µm sections and thaw mounted onto Indium tin oxide coated (ITO) glass slides. The sections were then vacuum packed and stored at −80 °C.

### 2.5. Matrix Application

In total, 19 mg of DHB matrix was dissolved in 15 mL of acetone and added to the bottom of the sublimation apparatus (Sigma-Aldrich, Gillingham, UK). An ITO glass slide containing the tissue disc section was secured to the flat surface within the top section of the sublimation apparatus. The top and bottom of the sublimation apparatus were then assembled using the O-ring seal and the vacuum was applied to seal. Once the vacuum had stabilized at 5 × 10^−2^ Torr, the top was filled with ice and the temperature was set at 180 °C. The sublimation process was stopped after 15 min.

### 2.6. Mass Spectrometry Imaging

All tissue sections in this study were imaged using a Bruker Autoflex III mass spectrometer. The instrument calibration was performed with CHCA matrix. The MALDI-MS images were acquired in positive ion mode focused on a range of *m*/*z* 120–1500 Da. The spatial resolution was set to 100 µm × 100 µm. Three separate sections from the same absorption experiment were imaged within the same batch.

### 2.7. Tissue Extraction and Sample Preparation

Each tissue disc was added to 30 mL of methanol: water (9:1, *v*/*v*) and homogenized. The donor circuit, acceptor circuit, and the solutions collected from rinsing the tissue and the system were each separately added 50:50 *v*/*v* to a solution of methanol: water (9:1 *v*/*v*). All of the solutions were then centrifuged for 5 min at 3000× *g*. The supernatant was collected for LC-MS/MS analysis. From a 50 µg/mL Atorvastatin stock solution, 6 standards and a blank were made ranging from 0–10 ug/mL. All standards and samples were prepared with an internal standard, 2.5 µg/mL of Atorvastatin-d5, matrix matched, and run-in triplicate.

### 2.8. LC-MS/MS

All LC-MS/MS experiments were performed using the Agilent 6420 triple quad mass spectrometer in negative ion mode. The analyzer was set to detect the product ion of Atorvastatin-d5 calcium salt (*m*/*z* 562 → *m*/*z* 458) and the product ion of Atorvastatin calcium salt (*m*/*z* 557 → *m*/*z* 453) in multiple reaction monitoring (MRM) mode. An Agilent EclipsePlusC18 RRHD 1.8 µm 2.1 × 50 mm column was used. The mobile phase consisted of water: ACN: methanol (41:19:40, *v*/*v*/*v*) with 0.005% formic acid and was used in isocratic mode at a flow rate of 0.275 mL/min.

### 2.9. Data Analysis

MALDI-MS data were processed using the flexImaging software from Bruker Daltonics. For the LC-MS/MS data, the chromatographic peaks for Atorvastatin and Atorvastatin-d5 were integrated and processed using the Agilent MassHunter Quantitative Analysis Version 8.09 software. This software was also used to create the calibration graph from the standards and calculate the concentration of all the samples. The back-calculations from software generated concentrations were performed manually.

The apparent permeability coefficient for transport across porcine intestinal tissue (*P*_app,pig_) was determined for each drug absorption experiment performed in the study [12]. The following equation was used to determine the *P*_app,pig_ value for each experiment:

(1) $$P_{\mathrm{app},\;\mathrm{pig}}=\frac{dc}{dt}\times \frac{V}{A\times C_{0}}\;\left(\frac{cm}{s}\right)$$

where *dc*/*dt* is the change in the acceptor concentration calculated using the final concentration in the acceptor circuit divided by the length of the experiment (6 h), *V* is the volume in the donor circuit (30 mL), *A* is the exposed surface area (0.785 cm^2^), and *C*_0_ is the initial concentration of Atorvastatin in the donor circuit (500 µg/mL).

## 3. Results and Discussion

### 3.1. QV600 LLI Adaptation

**Figure 1:**
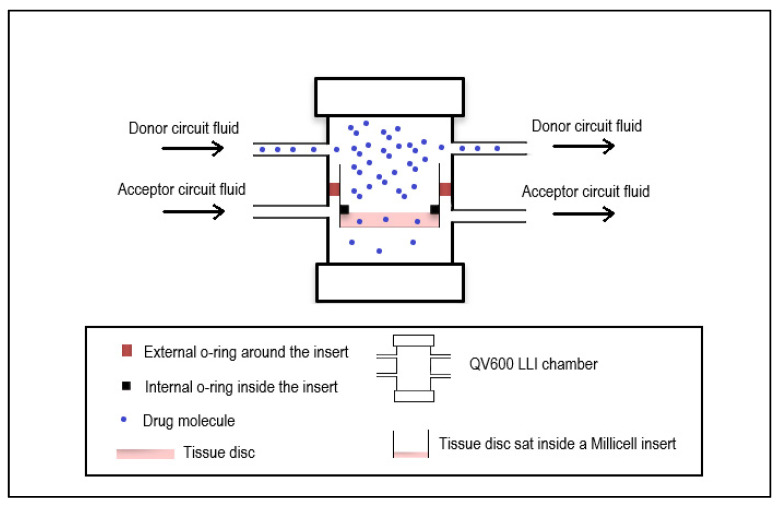
Adaptation of the QV600 LLI (Kirkstall Ltd.) microfluidics cell culture chamber to hold ex vivo tissue.

The QV600 LLI system was adapted to hold an ex vivo intestinal tissue disc in a Millicell insert without allowing the donor and acceptor circuit to interact (Figure 1). A complete barrier between these circuits was essential as any leakage of the donor circuit into the acceptor circuit would give a false representation of the movement of Atorvastatin through the tissue. The Atorvastatin would then have to travel through the intestinal tissue in order to reach the acceptor circuit. The intestinal tissue was cut into a disc using a biopsy punch that was slightly larger than the bottom of the insert. Thus, the tissue disc would cover the mesh layer at the bottom of the insert entirely. To prevent any movement or shifting of the tissue disc during the experiment, the tissue disc was fixed to the mesh and in addition, a silicon O-ring was fitted into the insert and placed on top of the tissue to seal off the edges as they were the most susceptible to movement. The original set-up of QV600 LLI with cells advertised by Kirkstall Ltd. allowed for free movement between the two circuits. With the adaptation reported here for use with ex vivo tissue, the system was more susceptible to pressure build-ups when following the original set-up guidance which would lead to leaks between the two circuits. A different set-up method was established which reduced the pressure build-up during the initial system set-up and prevented leakages from occurring. The system was run with the flow rates in the donor and acceptor circuits calculated to be 0.2 mL/min and 0.1 mL/min, respectively.

During the adaptation, development, and testing of the QV600 LLI, previously snap-frozen tissue was used. Therefore, there was no need to assess the viability of the tissue as this was lost during the freezing process. Thus, the experiment time was based upon the highest estimate for intestinal transit time which ranges between 3–6 h in the small intestine specifically [26]. Therefore, the experiments were performed for 6 h to ensure that the drug exposure time didn’t exceed what it would be in a whole organ. This also will allow for time point studies to be conducted using sampling ports to assess how drug absorption is affected over time. Once viable tissue is introduced into the system, the viability of the tissue will need to be assessed. Atorvastatin is rapidly absorbed after administration with a peak plasma concentration reported between 1–2 h which would make the study of Atorvastatin absorption appropriate for this set up as other ex vivo models have reported tissue viability up to 3 h [27].

### 3.2. Development of MALDI MSI for the Detection of Atorvastatin

A MALDI MS method was developed to profile the protonated molecule, sodium, and potassium adducts of Atorvastatin at *m/z* 559, *m/z* 581, and *m/z* 597, respectively, in positive ion mode. Initially, several different MALDI matrices were trialled for the detection of Atorvastatin, including CHCA. The optimal matrix was found to be DHB. Atorvastatin was dissolved in methanol: water (9:1, *v*/*v*) to create a series of known standards to test the sensitivity of the profiling method. The method was continuously improved to detect the three Atorvastatin-related peaks in prepared standards as low as 0.01 mg/mL. The MALDI MS profiling method was then converted into a MALDI MS imaging method using flexControl version 3.4 software.

The MALDI MS imaging method was then used to image 0.5 µL spots of known Atorvastatin standards that had been pipetted onto 14 µm sections of untreated duodenum intestinal tissue. The imaging method and matrix application were optimised to increase the signal from the protonated molecule, sodium, and potassium adducts of Atorvastatin when in the presence of tissue.

Once the imaging method was optimised, it was then ready to use for the imaging of tissue sections acquired from drug absorption experiments.

### 3.3. Development of LC-MS/MS for the Detection of Atorvastatin

An LC-MS/MS method was developed in MRM mode for the detection of Atorvastatin in tissue and liquid samples collected from the initial drug absorption experiment in the QV600 LLI. Matrix matched standards were prepared using the deuterated internal standard Atorvastatin-d5 to create a calibration graph for the quantification of Atorvastatin in the tissue discs and each sample collected from the experiment. The samples collected were the donor circuit solution, the acceptor circuit solution, the solution used to rinse the tissue disc, the initial system rinse in 70% methanol, and the final system rinse in PBS. The amount of Atorvastatin in each sample was quantified and used to establish a mass balance. The mass balance was calculated to ensure that an acceptable amount of the drug had been recovered in order to be representative of the drug movement through the system. Acceptable mass balances were considered to be between 85–115% for the purpose of this study.

The tissue discs were rinsed to remove any excess Atorvastatin that had not absorbed into the tissue. The final system rinse was collected to ensure that the QV600 LLI was considered analytically clean for the next experiment and also, to confirm that all the drug had been recovered from the system by the system rinse in 70% methanol. The collection of these samples was essential for the calculation of the mass balance, although they provide no context to the movement of the drug.

Due to matrix effects from the DMEM, an extensive matrix matched formula was developed for the preparation of the samples. Despite this, matrix effects were still experienced when the drug had originally dissolved in DMEM such as in the acceptor circuit. Therefore, an additional set of calibration standards were made using a stock solution in which Atorvastatin had been dissolved in DMEM prior to matrix matching the standards.

In addition to performing a mass balance, the concentration of Atorvastatin in the acceptor circuit was calculated to determine the amount of drug that travelled through the tissue disc and thereby, accumulated in the acceptor circuit.

### 3.4. Removal of Muscle-Serosal Layer

**Figure 2:**
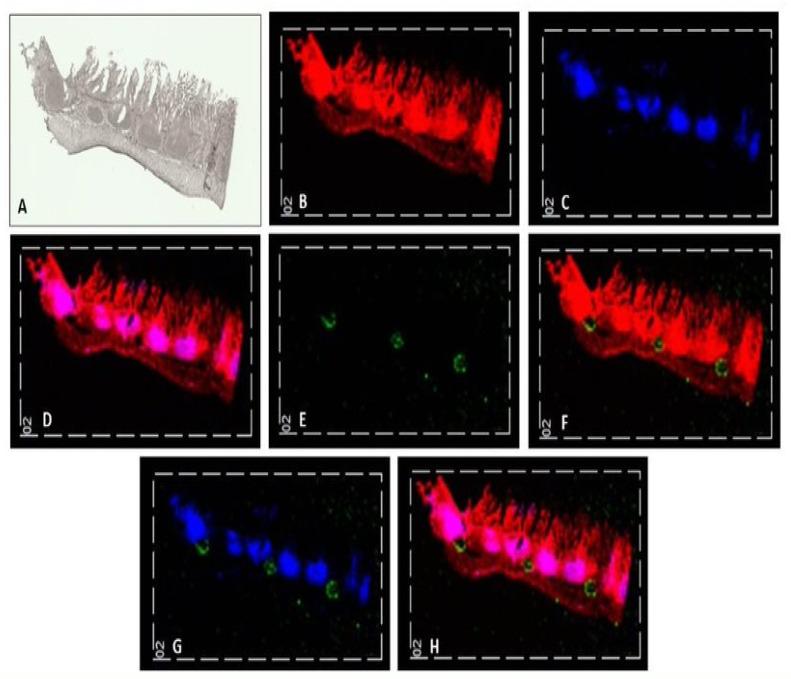
Using the QV600 LLI, intact porcine small intestinal tissue was treated with 0.5 mg/mL Atorvastatin over a 6 h period to investigate drug absorption. (**A**) A scanned image of the intestinal tissue section taken using a Super Coolscan 5000 ED Film Scanner with the apical layer facing upwards. (**B**) A MALDI-MS image showing cholesterol [Chol+H-H_2_O]^+^ at *m/z* 369 in red. (**C**) A MALDI-MS image showing the Peyer’s patches at *m/z* 389 in blue. (**D**) A MALDI-MS image showing cholesterol [Chol+H-H_2_O]^+^ at *m/z* 369 in red and Peyer’s patches in blue; overlapping ions are shown in pink. (**E**) A MALDI-MS image showing the sodium adduct of atorvastatin at *m/z* 581 in green. (**F**) A MALDI-MS image showing the sodium adduct of atorvastatin at *m/z* 581 in green and cholesterol [Chol+H-H_2_O]^+^ at *m/z* 369 in red. (**G**) A MALDI-MS image showing sodium adduct of atorvastatin (*m/z* 581) in green and Peyer’s patches (*m/z* 389) in blue. (**H**) A MALDI-MS image showing the sodium adduct of atorvastatin at *m/z* 581 in green, cholesterol [Chol+H-H_2_O]^+^ at *m/z* 369 in red and Peyer’s patches in blue; overlapping ions are shown in pink.

The muscle-serosal layer was left intact for the initial imaging drug absorption experiment to aid the visualisation of the distribution of the Atorvastatin within the section when Peyer’s patches are present. The experiment ran for 6 h with 0.5 mg/mL Atorvastatin passing over the surface of the apical layer of the duodenum intestinal tissue. The tissue discs from this experiment were cryosectioned and imaged using an optimised MALDI MS imaging method. The images generated from this experiment were reproducible in the three sections that were taken from one of the tissue discs, as shown in Figure 2.

The distribution of *m/z* 369 highlighted the whole tissue section, showing unique characteristics of the section which can be identified by comparing Figure 2A,B. The distribution of *m/z* 389 outlines a collection of lymphatic vessels within the tissue known as Peyer’s patches. These two ions were overlaid with *m/z* 581, the sodiated adduct of Atorvastatin as shown in Figure 2H. The identification of tissue structures in addition to the drug molecule in the tissue section using the MALDI MS imaging method provides invaluable spatial information that gives an insight into drug distribution within the tissue. The sodiated adduct of Atorvastatin is shown distributed in between the Peyers patches in the submucosal layer. In the image, the sodiated adduct appears to be pooled on top of the muscle-serosal layer and did not appear to have passed through the Peyer’s patches.

**Figure 3:**
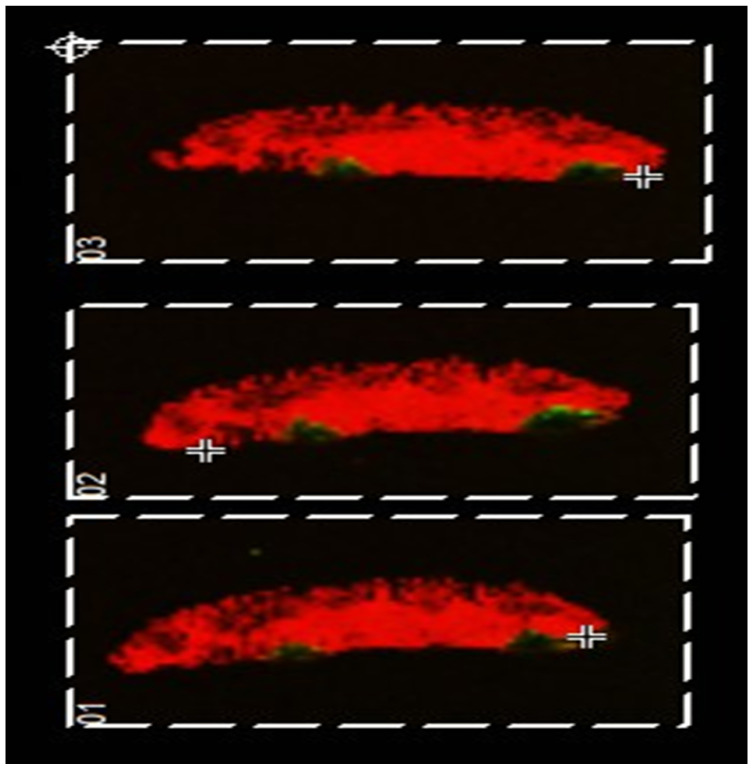
Using the QV600 LLI, porcine small intestinal tissue with the muscle-serosal layer removed was treated with 0.5 mg/mL Atorvastatin over a 6 h period to investigate drug absorption. A MALDI-MS image was generated showing cholesterol [Chol+H-H_2_O]^+^ at *m*/*z* 369 in red and protonated molecule of atorvastatin at *m*/*z* 559 in green.

The drug absorption experiment was repeated with the muscle-serosal layer carefully removed (as would be the case in the microfluidics device). The MALDI MSI images obtained are shown in Figure 3. Interestingly, here the ‘pooling’ of the drug (imaged in green) is not observed clearly demonstrating that the presence of the muscle-serosal layer in the absence of blood flow hinders the movement of Atorvastatin as might be expected.

Additionally observable in these data is the variability of the tissue structure of sections taken from the duodenum of the same animal. In Figure 2, a number of Peyer’s patches can be seen; however, in Figure 3, none are observable. This indicates that care must be taken when selecting tissue for the chambers as there is high variability even within the same region of the small intestine.

### 3.5. The Addition of Polysorbate 80 to the Adapted QV600 LLI

A series of experiments were performed as described in the methods section iii with the muscle-serosal layer removed. The experiments varied by the addition of the refined excipient Polysorbate 80 provided by CRODA Inc. (Note: The specific concentrations and ingredients used in the refinement of the excipient are proprietary). An initial experiment was performed as a control with no excipient added to the donor circuit. Five additional experiments were performed with different volumes of the excipient added to the donor circuit; these consisted of 0.4% *v*/*v*, 0.8% *v*/*v*, 1% *v*/*v*, 1.5% *v*/*v*, and 2% *v*/*v* refined polysorbate 80.

**Figure 4:**
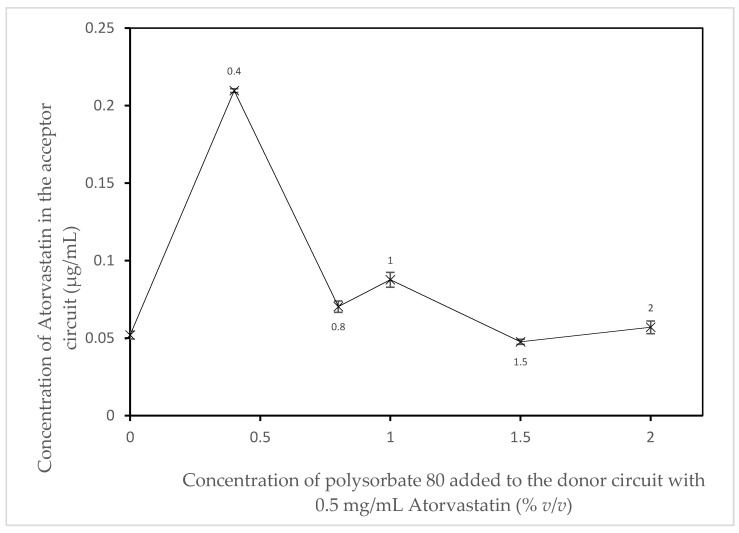
The effect of super refined polysorbate 80 LQ on the absorption of Atorvastatin in the duodenum section of ex vivo porcine small intestine (*n* = 3).

In Figure 4, an apparent increase in the absorption of Atorvastatin can be seen when 0.4 % *v*/*v* polysorbate 80 is added to the donor circuit. Above this concentration, there is no apparent effect of increasing amounts of polysorbate 80 are observed. This is in agreement with work first reported by Kaneda et al. [28]. They commented that there were two levels of surfactant effect on the absorption of drugs in the intestine. An absorption enhancing effect at low concentrations and a small inhibiting effect at higher concentrations. Interestingly, these effects have also been observed in this proof-of-concept study even though previously frozen tissue (i.e., active transport mechanisms and functional metabolising enzyme would not be expected to be present) was used. However, even with this system what has been termed “a solubility–permeability interplay” is still observable [29].

Drugs through viable porcine intestines using an Ussing chamber system [12]. Apparent permeability coefficients (P_app,pig_) were calculated and compared to known permeability coefficients determined in humans in vivo, P_eff,human_. The study reports a range of P_app,pig_ values for BCS class II drugs which span between 4.26 × 10^−^^6^ cm/s and 45.47 × 10^−^^6^ cm/s when in different donor circuit environments [12]. When comparing the P_app,pig_ values for BCS class II drugs with those determined in this proof-of-concept study, the values are of a similar order of magnitude as those reported. The relevant P_app,pig_ values calculated for this study are shown in Table 1. These data provide confidence that the adapted QV600 LLI has great potential for use in this field of work when viable tissue/3D cell culture models are used. It is important to highlight that the data provided in the proof-of-concept study have been acquired from ex vivo tissue that was previously frozen, whereas in the study of Arnold et al., viable tissue has been used [12]. Now that the proof-of-concept has proven that the QV600 LLI can be successfully adapted to house ex vivo and produce data comparable to permeation studies carried out by other ex vivo models, the next logical steps would be to repeat this study using ex vivo tissue that has not been previously frozen. The reason for this being that any P450 enzymatic activity within the small intestine would have been disabled during the freezing process. The presence of P450 enzymes in the small intestine contributes to the extensive metabolism of Atorvastatin prior to reaching systemic circulation, leading to the oral bioavailability of this drug reportedly as low as 14% [30]. From here, a direct comparison can be made between ex vivo tissue and the classical caco-2 cell line from within the QV600 LLI model. As the classical caco-2 cell line lacks P450 enzymes, it would be interesting to see the impact this could have on the permeation of Atorvastatin through the small intestine.

Due to the versatility of the QV600 LLI, this allows comparative studies to be performed within the same system as ex vivo human samples, cultured cells and organoids could be held in individual chambers. This would make for an interesting comparison between the apparent permeability coefficients to human in vivo data, thereby giving an insight into how the different types of models compare with each other in terms of their comparability to human in vivo studies.

## 4. Conclusions

This proof-of-concept study has demonstrated that the adapted QV600 LLI system is suitable for studying drug absorption in the gastrointestinal tract using ex vivo porcine tissue. Initial studies carried out using formerly frozen tissue showed the expected effects of a solubilizing excipient on drug absorption. Further work is currently in progress to repeat this study using fresh porcine tissue and further demonstrate the potential of the system for excipient studies where active transport mechanisms exist. Further modifications to the system by the incorporation of sampling ports will also allow for time point studies. The adaptation for ex vivo tissue allows the study of natural tissue structure using imaging techniques which is important to assess tissue variability. The use of mass spectrometry imaging allows not only the study of drug distribution and tissue pathology, but with further development will allow the quantification of the drug directly within the tissue sections.

## Figures and Tables

**Table 1:** The apparent permeability coefficient values for the passive absorption of Atorvastatin through ex vivo porcine tissue from a series of drug absorption experiments conducted using the modified QV600 LLI system in the presence of increasing amounts of the solubilizing agent polysorbate 80. (With corresponding mass balance data).

Concentration of Excipient Added to Donor Circuit (% *v*/*v*)	Concentration of Atorvastatin in Acceptor Circuit (µg/mL)	Apparent Permeability Coefficient (P_app,pig_) (10^−6^ cm/s)	Mass Balance (%)
0	0.052	0.234	95.2 ± 0.25
0.4	0.209	0.942	90.9 ± 0.67
0.8	0.074	0.336	95.9 ± 1.4
1	0.078	0.352	95.6 ± 2.6
1.5	0.046	0.207	94.3 ± 1.2
2	0.050	0.225	116.4 ± 1.1

Arnold et al. have conducted a study investigating the passive diffusion of eleven.

## Data Availability

Data is available for download from the Sheffield Hallam University Research Data Archive (https://shurda.shu.ac.uk/).
